# Regulatory Role of the Adipose Microenvironment on Ovarian Cancer Progression

**DOI:** 10.3390/cancers14092267

**Published:** 2022-05-01

**Authors:** Hussein Chehade, Roslyn Tedja, Harry Ramos, Tejeshwar Singh Bawa, Nicholas Adzibolosu, Radhika Gogoi, Gil Mor, Ayesha B. Alvero

**Affiliations:** 1 C.S. Mott Center for Human Growth and Development, Department of Obstetrics and Gynecology, Wayne State University, Detroit, MI 48201, USA; gm0971@wayne.edu (H.C.); roslyntedja@wayne.edu (R.T.); harryramos@wayne.edu (H.R.); tbawa@med.wayne.edu (T.S.B.); hc7424@wayne.edu (N.A.); radhikagogoi@wayne.edu (R.G.); gmor@med.wayne.edu (G.M.); 2 Karmanos Cancer Institute, Detroit, MI 48201, USA

**Keywords:** ovarian cancer, adipocyte, chemoresistance, metastasis, metabolic reprogramming

## Abstract

**Simple Summary:**

Adipocytes or fat cells are integral part of the ovarian tumor microenvironment. Secreted factors from adipocytes, as well as direct cell-to-cell interaction with ovarian cancer cells have been shown to directly support ovarian tumor progression. Elucidating the molecular pathways involved is crucial in the identification of relevant targets.

**Abstract:**

The tumor microenvironment of ovarian cancer is the peritoneal cavity wherein adipose tissue is a major component. The role of the adipose tissue in support of ovarian cancer progression has been elucidated in several studies from the past decades. The adipocytes, in particular, are a major source of factors, which regulate all facets of ovarian cancer progression such as acquisition of chemoresistance, enhanced metastatic potential, and metabolic reprogramming. In this review, we summarize the relevant studies, which highlight the role of adipocytes in ovarian cancer progression and offer insights into unanswered questions and possible future directions of research.

## 1. Introduction

Ovarian cancer remains the most common cause of death from gynecologic cancers worldwide [1]. In the US alone, it is estimated that 19,880 cases will be diagnosed and 12,810 deaths from this disease will occur in 2022 [2]. Despite major advances in the understanding of molecular mechanisms that support ovarian cancer progression stemming from innovations in -omics technologies [3,4,5,6,7,8] and development of novel and relevant model systems [6,7,9], the 5-year survival rate for ovarian cancer remains dismal at less than 50% [10].

Ovarian cancer is not a single disease, but instead represents a heterogeneous group of tumors with three major classifications based on site of origin: epithelial, sex-chord stromal, and germ cell. Epithelial ovarian cancer makes up about 85–90% of all cases. Still within the epithelial type, the disease is classified into four main sub-types: high- or low- grade serous, mucinous, endometrioid, and clear cell [8,11].

Ovarian cancer progresses within a microenvironment consisting of a supporting stroma of cancer-associated fibroblasts, adipocytes, vascular cells, and immune cells. Considerable crosstalk occurs between these different cell types, and various signaling mechanisms that support tumor growth and metastasis formation have been described [12,13,14]. To develop novel modalities to curtail ovarian cancer progression and improve patient survival, a better understanding of how the ovarian cancer microenvironment regulates tumor growth, chemoresponse, and propensity to disseminate is required.

Adipocytes are an integral part of the ovarian cancer microenvironment. The adipose-rich omentum has been shown to be the earliest site of ovarian cancer metastasis [15] and the most common site of residual disease [16] as well as recurrent disease [17,18,19,20]. Moreover, the extent of tumor debulking in the omentum and the response of adipose-associated metastatic disease to chemotherapy has been demonstrated to significantly impact patient survival [16,20], further highlighting the important contribution of this microenvironment.

In this review, we will discuss the role of adipocytes in ovarian cancer progression. We first define the adipose depots in the ovarian cancer microenvironment, then dissect the mechanisms by which adipocytes confer chemoresistance, promote metastasis formation, and support metabolic reprogramming leading to disease progression and patient demise. Finally, we discuss current translational efforts and future perspectives to curtail adipocyte-induced pro-tumor mechanisms, including specific targeting of treatment to this hydrophobic site.

## 2. Adipose Depots in the Ovarian Cancer Microenvironment

Ovarian cancer preferentially metastasizes via shedding into the peritoneal cavity and remains confined to this space [11,21,22]. Even in late-stage disease, metastasis to organs outside of the abdomen is very rare [23,24]. The peritoneal cavity is a space defined by the diaphragm, vertebrae, pelvic floor, and anterior abdominal muscles [25] and houses the stomach, spleen, liver, most of the small and large intestines, the omentum, and the mesentery. It is covered by a single layer of mesothelial cells attached to a basement membrane and supported by a thin layer of connective tissue composed of fibroblasts and collagen [26].

Within the peritoneal cavity, visceral adipose tissues surround the organs and are organized into specific depots: omental, mesenteric, retroperitoneal, and gonadal [27,28,29]. In addition to adipocytes, these adipose depots are comprised of other cell types such as pericytes, endothelial cells, and immune cells. The visceral adipose tissues in the peritoneal cavity are mostly the white adipose tissue type, which functions in the storage of excess dietary fat and its release during starvation or increase in energy demand [28,30]. More recent studies, however, have provided evidence of a more comprehensive function of white adipose, including appetite regulation, immunity, coagulation, control of vascular tone, and metabolic homeostasis [28,31,32,33,34,35].

The adipose-rich omentum has significant involvement in the progression of ovarian cancer [17,18] and is the most studied adipose tissue in the context of ovarian cancer progression. It is an apron-like visceral organ, which is derived from mesothelial cells from the yolk sac and develops as a visceral adipose tissue covering the organs of the peritoneum. It is lined by two-layers of mesothelial cells and has a rich vascular supply with a unique collection of cells such as adipocytes, endothelial cells, and “milky spots” comprised of leukocyte aggregates in perivascular areas [36]. In animal models, the omentum has been shown as the earliest site of ovarian cancer colonization [15]. Thus, the adipose-rich omentum has been associated with chemoresistance and metastasis formation, two key mechanisms that support ovarian cancer progression.

## 3. Adipose-Induced Mechanisms Promoting Chemoresistance in Ovarian Cancer Cells

Standard therapy for ovarian cancer includes surgical debulking followed by adjuvant chemotherapy with platinum and taxane agents resulting in ~80% complete response rate [37,38]. Unfortunately, 75% of these responders will relapse within 2 years and eventually develop chemoresistant recurrent disease, which rapidly becomes unresponsive to treatment [39]. Even with newer agents, randomized phase III trials of second-line therapy in patients with platinum-resistant high-grade serous ovarian cancer (HGSOC) have not shown a significant advantage over existing therapy with respect to progression-free survival (PFS) or overall survival (OS) [40,41,42,43,44].

The role of the adipose microenvironment in support of chemoresistance is underscored in studies, demonstrating that the response of adipose-associated metastatic disease to chemotherapy is proportional to survival [16,20]. Analysis of a cohort of 161 patients diagnosed with Stage III–IV high-grade serous ovarian cancer showed that chemotherapy response scores from omental disease showed a significant prognostic value for both overall and progression-free survival [20]. Similarly, analysis of 56 chemo-naive patients diagnosed with Stage III–IV epithelial ovarian, tubal, and peritoneal cancer showed patients with omental metastasis (*n* = 36) to have 43.4% 5-year survival rate, compared to 93.8% in those without omental involvement (*n* = 20) [45]. In this cohort, a significant difference in overall survival (*p* = 0.002) and progression-free survival (*p* = 0.036) between the omental metastasis-positive and metastasis-negative groups were noted [45].

Numerous studies have highlighted the mechanisms by which secreted factors from adipocytes can activate molecular pathways, leading to the acquisition of chemoresistance in ovarian cancer cells. Adipocytes secrete a myriad of cytokines, chemokines, hormones, and lipids. Leptin, for example, is a peptide hormone primarily produced and secreted by adipocytes in white adipose tissues in proportion to the size of fat depot and thus is directly proportional with obesity [46]. In addition to having an important role in the regulation of food intake and body mass [47,48], Leptin has been shown to directly contribute to chemoresistance in ovarian cancer cells. Survival database analysis of 1656 ovarian cancer patients identified those with high leptin (*n* = 108) and low leptin (*n* = 112) and showed that median leptin expression is associated with poorer prognosis in patients previously treated with platinum and paclitaxel/docetaxel [49]. These findings were replicated in vitro, wherein pre-treatment with exogenous leptin was shown to increase resistance to Cisplatin + Paclitaxel and Cisplatin + Taxotere in HO8910PM and OV-MZ-15 ovarian cancer cell lines [49]. Leptin has also been shown to upregulate the anti-apoptotic protein Mcl-1. Using OVCAR-3 human ovarian cancer cell line, Chen et al. demonstrated that exogeneous leptin activates several kinase pathways such as JAK2, Akt, and ERK, all contributing to the upregulation of Mcl-1 [50]. Interestingly, Leptin is one of the six-marker panel identified that can predict early-stage ovarian cancer, suggesting a possible role of Leptin in ovarian cancer initiation [51,52,53].

Bclxl, another anti-apoptotic protein, has also been shown to mediate adipocyte-induced chemoresistance. Bclxl is upregulated in CD44+/MyD88+ chemoresistant ovarian cancer stem cells compared to CD44−/MyD88− sensitive ovarian cancer cells. Interestingly, Bclxl expression is induced in CD44−/MyD88− cells in vitro by factors secreted by adipocytes, and this was associated with acquisition of resistance to Carboplatin [54]. A similar effect was observed in vivo, where Bclxl expression was higher in tumors isolated from metastatic implants localized in the adipose tissues. Using an ovarian cancer intra-peritoneal xenograft model, omental implants were shown to express higher levels of intra-tumoral Bclxl compared to ovarian tumors and metastatic implants in liver and mesentery [54]. In another study, conditioned media from both subcutaneous and visceral adipocytes were shown to stimulate the Akt pathway and induce Cisplatin resistance in several human ovarian cancer cell lines [55]. Yang et al. identified a lipid, arachidonic acid, as the main and direct mediator of this effect [55].

In addition to hormones, cytokines, and lipids, the adipose tissue can impact the tumor microenvironment and enhance resistance to chemotherapy through the release of exosomal microRNA. Yeung et al. reported that exosomal microRNA-21 (mir-21) from cancer-associated adipocytes and fibroblasts confer Paclitaxel resistance in OVCA432 and SKOV3 ovarian cancer cells both in vitro and in vivo. They identified Apoptotic protease activating factor-1 (APAF-1) as a novel target of mir-21 that mediates the observed Paclitaxel resistance [56]. APAF-1, together with Cytochrome C and dATP, forms the apoptosome enzyme complex, which cleaves and activates Caspase-9 and is therefore required to propagate mitochondria-initiated apoptosis [57].

Adipocytes have also been shown to promote chemoresistance indirectly. Co-culturing of SKOV3ip1 human ovarian cancer cells with human omental biopsies identified FABP4 as one of the 16 significantly altered proteins in the cancer cells [58]. The functional consequence of this was shown to be resistance to carboplatin, as inhibition of FABP4 with BMS309403 shifted IC_50_ to Carbpoplatin in PEO1 and PEO4 human ovarian cancer cell lines [58]. Another indirect way that adipocytes may promote chemoresistance is by remodeling the extracellular matrix, specifically by inducing overexpression of Collagen VI. Adipocyte-derived collagen VI has been shown to promote progression in a breast cancer model [59] and cultures of A2780 human ovarian cancer cells in collagen VI induced Cisplatin resistance [60].

**Figure 1:**
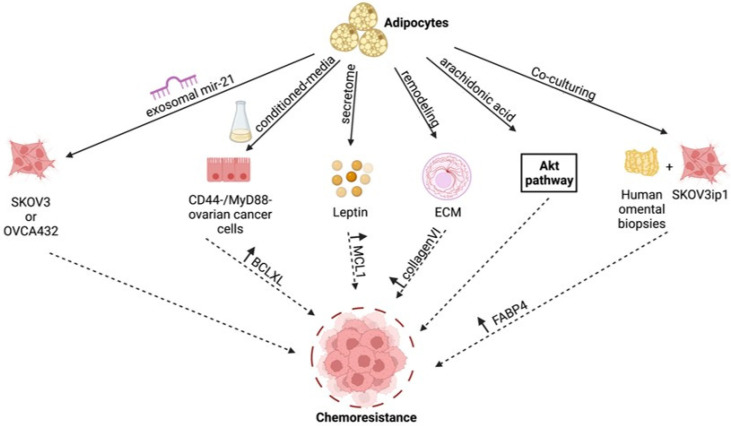
Adipose-induced mechanisms supporting chemoresistance. Figure summarizes the various molecular mechanisms unraveled in the studies described in text [46,49,50,51,54,55,56,57,58,59]. Created with Biorender.com (accessed on 1 April 2022).

Taken together, these studies highlight the molecular pathways induced by the adipose tumor microenvironment in ovarian cancer cells to promote chemoresistance. As summarized in Figure 1, many of these pathways are essential for tumor progression and are regulated by adipocyte-derived factors. These findings provide a new avenue for developing novel therapeutic approaches. Modulation of adipocyte-derived factors can be developed as a modality to prevent chemoresistance.

## 4. Adipose-Induced Mechanisms Promoting Metastatic Potential in Ovarian Cancer Cells

The role of adipocytes in metastasis formation has been reported in different solid tumors such as breast and prostate cancers, which preferentially to the adipose-rich site of the bone marrow [61,62,63,64]. However, unlike breast and prostate cancers that follow the classical pattern of hematogenous route of metastasis, ovarian cancer rarely metastasizes via this route and instead metastasizes via the transcoelomic route and mostly remains within the peritoneal cavity [19]. An intriguing question is whether adipocytes play a role in ovarian cancer metastasis formation. This question is relevant in understanding the mechanisms that promote carcinomatosis, especially as, as described above, the peritoneal cavity is surrounded by several adipose depots.

Ovarian cancer metastasis begins when single cells or multi-cellular spheroids shed from the primary tumor, are carried by peritoneal fluid or malignant ascites, and land on secondary sites [65]. This process has been divided into three distinct stages [19,66]: (1) detachment from the primary tumor, (2) migration, and (3) invasion and implantation to secondary site(s), each stage with its own, as well as shared, regulators and required pathways [19]. Adipocytes are known to influence these stages, and, in this section, we will review reported adipose-derived mechanisms that can support each step of the metastatic process.

### 4.1. Detachment from the Primary Location

Even though ovarian cancer presents with a unique mode of metastasis compared to other solid tumors, the process of epithelial–mesenchymal transition (EMT) remains to be a crucial step in its initiation [67,68]. The first step in this differentiation process is the loss of apicobasal polarity in the epithelial cells resulting from changes in the dynamics of adherent junctions (such as cadherins), tight junctions (including ZO-1, ZO-2, ZO-3, occludins, and claudins), and actin cytoskeleton (such as CK8 and CK18). In this subsection, we will review how adipocytes modify adherent and tight junctions and the cytoskeleton, which can initiate EMT.

The most described initiation step in EMT is the loss of E-cadherin, which is a main component of adherent junctions that maintain epithelial phenotype and cell-to-cell interaction [69]. Meta-analysis of 1562 ovarian cancer patients across 17 different studies showed that the loss of E-cadherin predicted poorer overall survival [66,70,71]. In addition, E-cadherin expression has been reported to be significantly lower in metastatic lesions compared to the primary tumor in ovarian cancer patients [71]. There are known soluble growth factors that induce E-cadherin loss in cancer cells. Some of these soluble factors are produced by adipose-rich tissues such as the hepatocyte growth factor (HGF) [72]. HGF has been reported to reduce the expression of E-cadherin, β-catenin, and caveolin-1 in HO8910 ovarian cancer cell line in vitro [73]. Transfection of CaOV3 and SKOV3 ovarian cancer cells with HGF prompted a change to a fibroblastic-like shape and activation of p70 S6 kinase [74]. This change in appearance correlated with increasing Snail expression, which was confirmed in vivo [74]. Another growth factor secreted by adipocytes is insulin-like growth factor-1 (IGF-1) [75]. In a study performed using ovarian cancer cell lines OVCAR5 and SKOV3 in vitro, IGF-1 was shown to inhibit E-cadherin expression via the PI3K/Akt/mTOR signaling pathway [76]. Additionally, fibroblast growth factor (FGF), which is also secreted by adipose tissue-derived stromal cells [77,78] has been shown to induce E-cadherin down regulation via PI3K/Akt/mTOR and MAPK/ERK in OVCAR4 and SKOV3 human ovarian cancer cells [79].

In addition to these soluble growth factors, cytokines that are secreted by adipocytes are also known to contribute to the initiation of EMT. One of these cytokines is interleukin-6 (IL-6) [80]. Treatment of SKOV3 and IGROV-1 ovarian cancer cells with IL-6 led to a E-cadherin to N-cadherin switch and the upregulation of mesenchymal markers Snail and Twist [81]. Another well-studied adipokine is interleukin-8 (IL-8) [82]. IL-8-treated SKOV3 and OVCAR3 ovarian cancer cell lines demonstrated decreased E-cadherin, increased β-catenin, and enhanced migratory capacity in trans-well assays [83]. IL-8-initiated EMT was also demonstrated in SKOV3 and A2780 cells and also shown to be regulated via the β-catenin signaling pathway [84]. Moreover, the inhibition of IL-8 signaling using neutralizing antibodies reduced migration and adhesion of SKOV3ip1 ovarian cancer cell line to human omental sections in vitro [15]. Taken together, these results demonstrate that IL-8 is able to support the three steps in ovarian cancer metastatic process.

### 4.2. Migration to Secondary Location

Following detachment from the primary tumor, ovarian cancer cells that have undergone EMT are now able to resist the selective pressure of anoikis and are poised to successfully create a secondary metastatic site. Anoikis is the activation of the apoptotic cell death pathway in cells upon detachment from its extracellular matrix (ECM) [85]. Anoikis resistance allows detached ovarian cancer cells to survive in the absence of ECM contact and migrate to secondary sites in the peritoneal cavity [67,86]. The adipose microenvironment has been shown to aid the process of migration by the production of several adipokines, growth factors, and hormones.

For instance, conditioned media derived from CD45−/CD31− adipose stromal cells, which are isolated from subcutaneous or visceral fat, was shown to activate the JAK2/STAT3 pathway via IL-6 and enhance migration in SKOV3 ovarian cancer cells [87]. Furthermore, exogenously added IL-6 was shown in a separate study to activate the JAK2/STAT3 pathway and promote migration in SKOV3 and OVCA433 ovarian cancer cell lines [88]. IL-8, as mentioned above, is an adipokine that can promote migration of ovarian cancer cells both in vitro and in vivo. Treatment with IL-8 induced migration, while knock-down of IL-8 inhibited migration of SKOV3 ovarian cancer cells in vitro. In vivo, neutralizing antibodies to both IL-8 and IL-6 prevented homing to adipose microenvironment in the SKOV3ip1 ovarian cancer xenograft model [15]. Still, interleukin-33 (IL-33), another adipokine [89] and part of the IL-1 gene family, has been shown to activate ERK signaling to promote migration and invasion in CAOV3 ovarian cancer cells [90]. Finally, MCP-1, which is produced by adipocytes, has been shown to induce migration and omental metastasis by binding to its cognate receptor CCR-2 on ovarian cancer cells and in turn activating the PI3K/AKT/mTOR pathway and its downstream effector HIF-1a and VEGF-A [91,92].

In addition to its effect on E-cadherin described above, HGF has also been shown to promote migration in several ovarian cancer cell lines in vitro [93]. Along the same line, neutralizing antibodies against HGF inhibited the migration of SKOV3 ovarian cancer cell line [94].

Leptin, as mentioned above, is an adipose-derived hormone. Exogenous treatment of leptin in SKOV3 and OVCAR3 ovarian cancer cells resulted in the activation of EKR1/2 and JNK1/2 and induced expression of MMP-7, -2, and -9, which are factors known to induce migration [95]. Similarly, in a separate study, leptin treatment was shown to induce migration and wound healing capacity in SKOV3 and HEY3 ovarian cancer cell lines [96].

### 4.3. Mesothelial Clearance and Invasion

Following the acquisition of anoikis resistance and successful migration to secondary sites, ovarian cancer cells must invade the mesothelial lining covering most of the peritoneal cavity. The first step in mesothelial clearance is believed to be the binding of ovarian cancer cells to extracellular matrices produced by mesothelial cells. These include hyaluronan, collagens type I and IV, laminin, and fibronectin [97]. CD44 expression in ovarian cancer cells is crucial in binding hyaluronan, and TNFα, which is secreted by adipocytes [98], has been reported to induce the expression of CD44 in ovarian cancer cells through activation of JNK [99]. The crucial role of CD44 in cancer metastasis has also been highlighted in other cancers, including breast cancer. Increased expression of CD44 has been shown to enhance adhesion capacity of breast cancer cells through HGF signaling [100].

Upon attachment, ovarian cancer cells need to invade through the mesothelial layer, which covers most of the peritoneal cavity. This requires the expression of matrix proteases such as MMPs. MMP2 has been reported to be upregulated following exposure to leptin in different types of cancer cells such as breast [101] and esophageal cancers [102]. In ovarian cancer, leptin was shown to induce MMP7 (known to degrade collagen type IV, laminin, and fibronectin) and promote invasiveness by activating ERK and JNK pathways [103].

**Figure 2:**
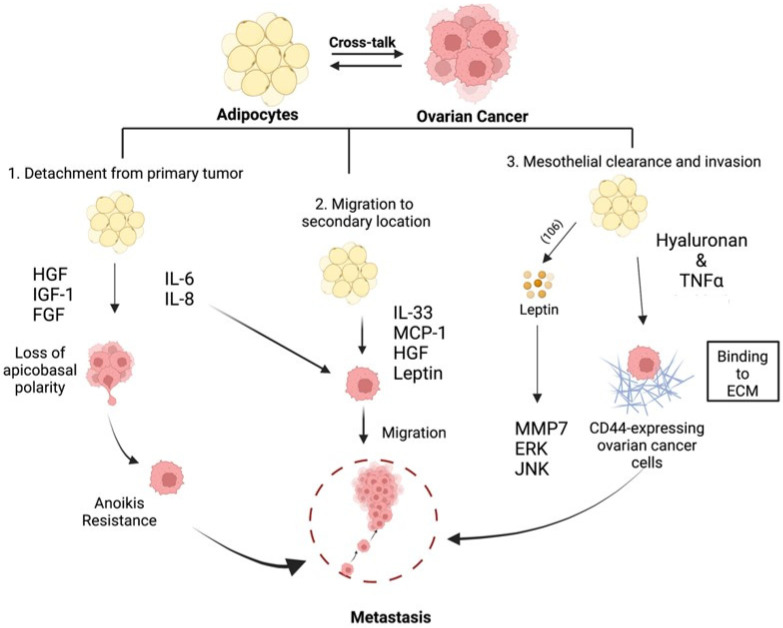
Adipose-induced mechanisms supporting metastasis formation. Adipose-derived factors are able to promote EMT, anoikis resistance, migration, and invasiveness to support all facets of metastasis formation [14,67,68,74,75,77,79,80,81,82,83,84,85,86,91,92,93,94,96,97,99,100,104]. Created with Biorender.com (accessed on 1 April 2022).

We postulate that adiposity can influence the tumor microenvironment leading to epigenetic modifications that promote ovarian cancer migration and invasion and that modification of this microenvironment could prevent metastasis. This hypothesis is supported by studies showing that pre-treatment of adipocytes with the hypomethylating agent guadecitabine abolished adipocyte-induced ovarian cancer cell migration and invasion [104]. Taken together, these studies demonstrate that the modulation of the adipose microenvironment can impact all stages of the metastatic process (Figure 2).

## 5. Adipocytes and Metabolic Reprogramming in Ovarian Cancer

Metabolic plasticity enables cancer cells to adapt to the ever-changing conditions in the tumor microenvironment during cancer progression. Uncontrolled proliferation, evasion of apoptosis to resist chemotherapy and the stepwise process of metastasis formation are processes that demand a significant amount of cellular energy and the generation of substrates for biosynthetic pathways. The ability to produce enough energy and metabolites that can support these processes in the context of a tumor microenvironment that constantly presents constraints in nutrition and oxygen availability requires a significant amount of metabolic plasticity. In order to sustain rapid proliferation and metastatic processes, cancer cells are required to fine-tune their metabolism and adapt to their new microenvironment. As stated above, during ovarian cancer progression, a main site of metastasis formation is the adipose-rich omentum. This microenvironment is rich in fatty acids and two early events have been identified in the crosstalk between ovarian cancer cells and adipocytes. First is the induction of lipolysis in the adipocytes and second is the enhanced uptake and utilization of the released fatty acids by the ovarian cancer cells. Co-culture of SKOV3ip1 ovarian cancer cells with omental or peritoneal adipocytes induced lipolysis in the adipocytes and transfer of the released fatty acids to the cancer cells [15]. At the same time, in the cancer cells, an increase in the fatty acid chaperone FABP4 was observed as well as an increase in β-oxidation and cancer cell growth [15,58]. These responses are essential, as they sustain membrane biosynthesis during rapid proliferation and provide sufficient energy source during metabolic stress.

Another molecular mechanism by which adipocytes alter ovarian cancer cell metabolism is by the activation of Salt-inducible kinase 2 (SIK2). SIK2 was shown to be upregulated in adipose tissue-associated ovarian cancer cells and demonstrated to promote ovarian cancer cell fatty acid oxidation. Adipose-induced SIK2 activation promoted fatty acid oxidation by phosphorylation of acetyl Co-A carboxylase and upregulation of CPT1 and, in parallel, activates the PI3K/Akt axis to support cancer cell proliferation and survival [105]. In a separate study, SIK2 was shown to enhance fatty acid and cholesterol synthesis in ovarian cancer via the SREBP1c/FASN and SREBP2/HMGCR axes, respectively [106].

Increased lipid uptake and fatty acid oxidation can also occur downstream of the apelin-APJ axis. Apelin is an adipokine, and its receptor APJ is upregulated in several ovarian cancer cell lines [107]. Adipocyte-derived factors can increase lipid uptake in ovarian cancer cells via the apelin–APJ signaling and promote fatty acid oxidation via the AMPK-CPT1 [107]. Inflammation represents another type of adipocyte-derived signal that can increase lipid uptake. Indeed, it has been shown that inflammatory cytokines, such as IL17A, increase lipid uptake in ovarian cancer cells via the STAT3/FABP4 axis, leading to enhanced growth both in vitro and in vivo [108].

**Figure 3:**
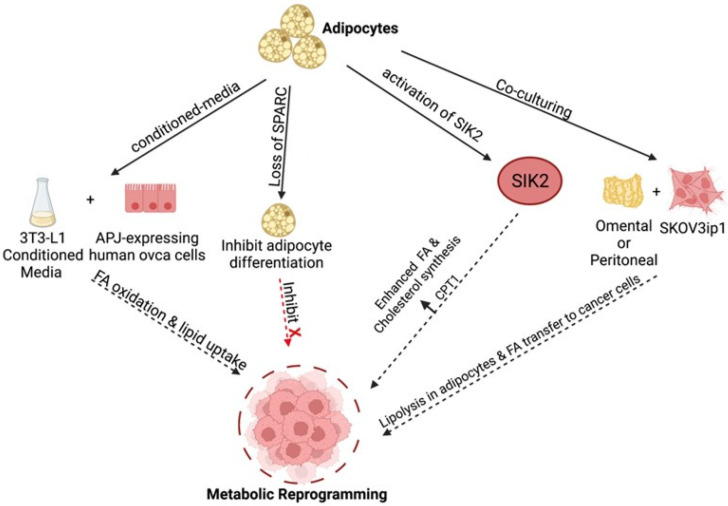
Adipose-induced mechanisms supporting metabolic reprogramming. Various adipose-derived factors promote fatty acid (FA) uptake and enhanced β-oxidation in ovarian cancer cells through various pathways to fuel tumor growth [14,57,106,107,108,110]. Created with Biorender.com (accessed on 1 April 2022).

Based on these observations, we postulate that metabolic adaptation, which is essential for tumor survival, is highly influenced by signals originated from malignant adipocytes (Figure 3). Although still not well understood, there are differences in the type of factors secreted by adipocytes from healthy individuals compared to those diagnosed with cancer. Analysis of minimal residual disease from patients with high-grade serous ovarian cancer demonstrated a molecular signature resembling both tumor-initiating cells and an adipose-like gene signature [109]. Transcriptomic analysis of paired pre- and post- neoadjuvant chemotherapy samples showed that ovarian cancer minimal residual disease has elevated expression of classical tumor-initiating cell markers and genes required for lipid metabolism. Genes required for anabolic and catabolic lipid processing were upregulated and associated with chemoresistance and mesenchymal phenotype [109]. Are the changes in the adipocytes due to the presence of cancer cells? Or are the changes in the adipocytes (e.g., inflamed adipocytes) the main regulator of malignant adaptation? Either way, it is evident that adipose tissues can influence malignant transformation and provides signals enhance metabolic transformation. Furthermore, malignant adipose tissue can be a target for cancer prevention. Indeed, John et al. demonstrated that inhibition of adipocyte differentiation by the loss of SPARC curtailed ovarian cancer growth, their migration, invasion, and homing to adipose-rich niches as well as inhibited ovarian cancer cell metabolic reprogramming [110].

## 6. Perspectives, Translational Aspects, and Future Directions

The studies summarized above highlight the myriad of molecular pathways initiated by adipocytes and activated in ovarian cancer cells to support tumor progression. It should be noted that this can be further confounded with other patient demographic factors such as menopausal status and obesity [111,112,113]. Hormones and body mass index (BMI) can regulate adipokine secretion and impact tumor vascularization as well as phenotypes of infiltrating immune cells. Further, in addition to these molecular barriers to treatment, adipose tissue can also impose a “structural barrier”, which can limit the efficacy of therapies. This is especially true in the context of obesity, wherein stiffened extra-cellular matrix [114] can curtail the efforts in sustaining the required amount of chemotherapy necessary to induce cancer cell death. A high degree of fibrosis, for example, has been observed in the adipose microenvironment of in vivo models of pancreatic cancer [115]. As such, the development of novel drug delivery platforms that can enhance adipose permeation may be of value in the quest to improve ovarian cancer patient survival. An example is the use of collagenase conjugated to gold nanoparticles. In a breast cancer model, enhanced metformin penetration and cytotoxic activity was observed upon encapsulation in gold nanoparticles functionalized with collagenase [116]. It is possible that nanoparticles engineered to target adipose tissues for the treatment of obesity [117] may be of value in cancer therapy.

In recent years, there is an emerging consensus that EMT is a multi-step process, which generates distinct cellular states between the epithelial phenotype start point and the mesenchymal phenotype endpoint [118]. Cancer cells with an intermediate phenotype between the epithelial and mesenchymal spectrum are referred to as E/M hybrid cells and co-express both epithelial and mesenchymal markers [119,120]. These cells have been described in circulating tumor cells as well as primary tumors from prostate, lung, and breast cancer patients [121,122,123]. In breast cancer, for example, CD104+/CD44hi breast cancer cells have been shown to co-express both epithelial (Krt8) and mesenchymal (N-cadherin, vimentin, Twist, etc.) markers, lacking plasticity but highly tumorigenic [119]. In ovarian cancer, characterization of 43 human ovarian cancer cell lines identified a majority of them to belong to the intermediate epithelial and intermediate mesenchymal phenotype, and sub-classification of ovarian cancer patients into an EMT spectrum correlates with progression-free survival [124]. Can the adipose microenvironment aid in the generation of ovarian cancer cells in the E/M hybrid state? We found initial evidence in our unpublished data that HGF, a factor secreted by adipocytes, can remodel focal adhesion and the cytoskeleton to generate ovarian cancer cells that co-express both epithelial and mesenchymal markers and generate cells with acquired anoikis resistance and enhanced invasiveness.

The combined actions and effects of oncogenes and mutated tumor suppressors, oxygen and nutrient availability, cell composition of the surrounding supporting stroma, and exogeneous therapies lead to a dynamic and heterogeneous intra-tumoral metabolism. Specifically, for ovarian cancer, this tumor microenvironment is almost always limited to the peritoneal cavity, and mostly associated with adipose-rich niches. If adipose-induced metabolic reprogramming is induced by secreted factors, then cell-to-cell contact may not be required for some of these mechanisms. Indeed, enhanced lipid metabolism has been observed in ovarian cancer cells suspended in ascites [125]. A robust cell-to-cell crosstalk mechanism is the secretion of exosomes, and an example of this has been mentioned above as an adipose-derived mechanism that promotes chemoresistance. It is therefore highly likely that mechanisms that promote EMT and metabolic reprogramming can also be due to exosomal cargo. This would mean that homing and direct contact with the adipocytes may not be necessarily required to acquire these adipose-initiated pro-tumor mechanisms. It should be noted that circulating exosomes are modulated by hormones and adiposity [126,127,128,129], which can be reflected in the tumor microenvironment.

## 7. Conclusions

**Figure 4:**
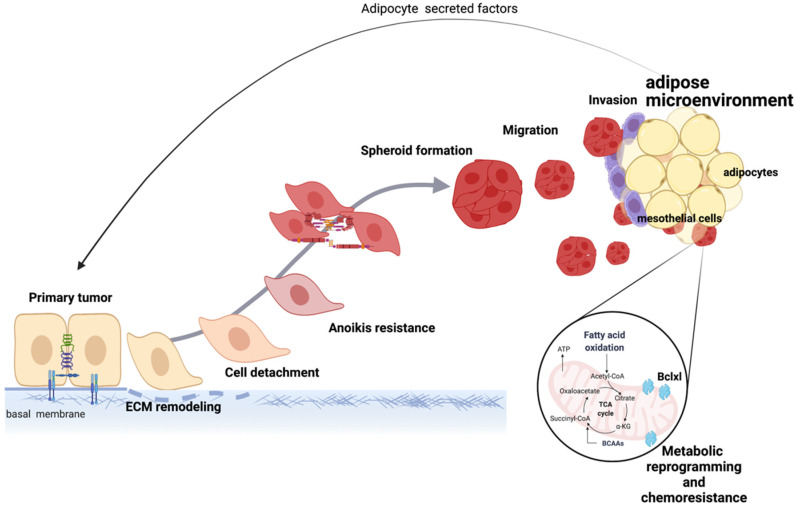
Mechanisms induced by the adipose microenvironment support ovarian tumor progression. Secreted factors from adipocytes and direct cell-to-cell contact between adipocytes and ovarian cancer cells lead to EMT, metabolic reprogramming, and acquisition of chemoresistance, which collectively support disease progression. Created with Biorender.com (accessed on 1 April 2022).

In summary, the adipose microenvironment is a preferred site for ovarian cancer metastasis and is the most common site of both residual and recurrent disease. Current evidence supports the role of the adipose microenvironment in multiple facets of ovarian tumor growth such as ovarian cancer cell proliferation, migration, chemoresistance, and metabolic adaptability (Figure 4). Although these pathways have been elucidated, many important questions remain to be answered, most importantly the translational aspect of these findings. Targeting the primary pathways in the crosstalk between adipocytes and ovarian cancer cells is currently an endeavor of multiple research laboratories around the world.
